# Age-Stratified Imaging Selection in Langerhans Cell Histiocytosis: Towards a Clinical Decision Framework

**DOI:** 10.3390/jcm15072568

**Published:** 2026-03-27

**Authors:** Brandon Gettleman, Sumin Jeong, Kole Joachim, Michael Fice, Adrian Lin, Casey Abernethy, Ezekiel Dingle, Amanda Perrotta, Alexandra E. Richards, Nicholas M. Bernthal, Alexander B. Christ

**Affiliations:** 1 Department of Orthopaedic Surgery, University of California, Los Angeles, CA 90095, USA; bgettleman@mednet.ucla.edu (B.G.); aerichards@mednet.ucla.edu (A.E.R.); nbernthal@mednet.ucla.edu (N.M.B.); 2 David Geffen School of Medicine, University of California, Los Angeles, CA 90095, USA; suminjeong@mednet.ucla.edu (S.J.); kjoachim@mednet.ucla.edu (K.J.); adrianlin@mednet.ucla.edu (A.L.); cabernethy@mednet.ucla.edu (C.A.); edingle@mednet.ucla.edu (E.D.); aperrotta@mednet.ucla.edu (A.P.); 3 Department of Orthopaedic Surgery, Greater Los Angeles VA Medical Center, Los Angeles, CA 90073, USA

**Keywords:** Langerhans, PET imaging, skeletal survey, bone scan, LCH

## Abstract

**Background/Introduction**: Standardized staging imaging for Langerhans Cell Histiocytosis (LCH) is lacking. This study aims to identify patient and disease factors that influence imaging selection and propose a clinical decision algorithm that supports more deliberate, radiation- and cost-conscious use of imaging. **Methods**: A retrospective review of patients with biopsy-proven LCH, evaluated at a single institution, was conducted from 2001 to 2025. Age, sex, and presenting location at diagnosis were compared across imaging groups (Positron emission tomography/computed tomography [PET/CT], bone scintigraphy, and skeletal survey). Disease extent (unifocal, multifocal, multisystem) was summarized according to the imaging modality each patient received. **Results**: The cohort included 78 patients: 30 PET/CT, 11 bone scans, and 37 skeletal surveys. Median age differed significantly by modality: PET/CT 34 years (interquartile range [IQR] 8–56) vs. bone scan 13 years (3–37) vs. skeletal survey 7 years (2–14) (*p* < 0.001). Imaging selection varied significantly by anatomic location (*p* = 0.016): bone lesions were assessed with skeletal survey (52.8%), PET/CT (28.3%), and bone scan (18.9%). All pulmonary cases received PET/CT imaging. Six of the 78 patients had multisystem disease, and all six had been staged with PET/CT (Fisher’s exact *p* = 0.006, PET/CT vs. skeletal survey; Bonferroni-adjusted *p* = 0.018). **Conclusions**: In addition to age, the initial presentation site influences the choice of imaging modality. Given the higher radiation burden and cost of PET/CT, further research is needed to determine whether this observed practice pattern translates to improved clinical outcomes.

## 1. Introduction

Langerhans cell histiocytosis (LCH) is a rare disorder resulting from the aberrant proliferation of antigen-presenting dendritic cells. Historically, LCH has been considered a childhood disease, with an annual incidence of 4.5 per one million children under 15 years old; however, it is also an important diagnosis to consider in older individuals, with a reported annual incidence of 1–2 per million in those over 15 years old.

While commonly thought of as a bone-related pathology, LCH can present in three distinct syndromes with indistinguishable histology: eosinophilic granuloma (EG), Hand-Schüller-Christian disease, and Letterer-Siwe disease [1,2]. The most common of the syndromes is EG, a benign condition typically limited to the bone or lung and characteristically found in children under 15 years of age. Lesions of EG, in over 50% of cases, present as lytic bone lesions, which can be treated with intralesional curettage surgery, and at times even spontaneously regress, especially after biopsy [3,4]. Hand-Schüller-Christian disease is the second most common form, accounting for approximately 10–20% of cases [5]. This pathology typically affects children under 10 years old and often presents with a rash and lesions to the skull and pituitary gland [5,6,7]. The rarest and most aggressive form of LCH is Letterer-Siwe (LS), a systemic disease commonly diagnosed in children under 2 years old, including lesions to the skull, peripheral bones, liver, spleen, and gastrointestinal tract, often leading to mortality at a young age [5,8]. Prognostic factors for these patients have been associated with age, timing of multisystem organ involvement, and initial response to chemotherapy during the induction phase [9,10,11,12]. Given the heterogeneity of disease presentations ranging from localized bone lesions to life-threatening multisystem involvement, accurate staging and disease extent assessment are critical for determining prognosis and guiding appropriate treatment decisions.

The diagnostic algorithm employed to assess LCH disease burden properly and the best treatment plan relies on the optimal selection of advanced imaging [13,14]. Current modalities include skeletal surveys, radionuclide bone scans, positron emission tomography/computed tomography (PET/CT), PET/Magnetic resonance imaging (PET/MRI) and whole-body MRI (WBMRI). PET/CT demonstrates greater sensitivity than bone scintigraphy for detecting early metastatic bone disease through metabolic assessment of both osteoblastic and osteolytic lesions, whereas bone scintigraphy primarily detects osteoblastic activity and may fail to identify purely osteolytic disease [15,16,17,18,19]. Similarly to bone scintigraphy, a skeletal survey has limited sensitivity for detecting metastatic disease, as it only assesses skeletal abnormalities [20]. Radiation exposure differs substantially between modalities, with PET/CT and bone scans delivering comparable effective doses of 4–7 millisieverts (mSv) and 4–6 mSv, respectively, and significantly lower exposure for skeletal surveys at 0.2–0.3 mSv [21,22,23,24]. Cost differences are equally substantial, with PET imaging representing the most expensive option at $900–1400 per study, compared to bone scintigraphy at $300–600 and a skeletal survey at $150–400 [25,26,27].

Currently, standardized staging imaging for LCH is lacking, which may contribute to a decreased ability to detect disease more efficiently, guide management effectively, and avoid delays in care. Therefore, this study aims to identify patient and disease factors that influence selection among three commonly used staging modalities at our institution (skeletal survey, radionuclide bone scintigraphy, and PET/CT) and to propose a clinical decision algorithm that supports more deliberate, radiation- and cost-conscious use of imaging. Because imaging modality selection varies widely and is influenced by clinical judgment, our data reflects practice patterns rather than controlled comparisons of diagnostic performance.

## 2. Materials and Methods

### 2.1. Study Design and Data Collection

We performed a retrospective chart review of all patients with LCH who underwent initial staging imaging between August 2001 and February 2025 at our institution. Patients were included if they had histologically confirmed LCH, initial staging imaging performed at our institution, and complete medical records available for review. Medical records were systematically reviewed to extract demographic, clinical, and imaging data. Collected variables included patient age at diagnosis, sex, disease extent at diagnosis, imaging modality utilized, and additional disease sites detected on imaging. The primary research question was whether patient age, anatomic site of initial presentation, and disease extent at diagnosis were associated with imaging modality selection and multisystem disease detection in LCH. For analysis, patients were categorized according to the initial staging modality used (skeletal survey, radionuclide bone scintigraphy, or PET/CT), and comparisons were performed to evaluate real-world practice patterns rather than diagnostic test performance. Any reference of PET refers to PET/CT.

### 2.2. Disease Extent Classification and Analysis

The extent of disease at diagnosis was systematically classified into three distinct categories: unifocal disease (single lesion at one anatomic site), multifocal single-system disease (multiple lesions confined to one organ system), and multisystem disease (involvement of two or more organ systems). This classification was performed based on clinical presentation, biopsy results, and imaging findings at the time of initial evaluation.

### 2.3. Statistical Analysis

Descriptive statistics were used to summarize patient characteristics and outcomes. Continuous variables were reported as medians with interquartile ranges. Normality of age distributions within each imaging group was assessed using the Shapiro-Wilk test. Because non-normality was identified in the skeletal survey group (W = 0.725, *p* < 0.0001) and Bartlett’s test confirmed significant heterogeneity of variances (W = 19.04, *p* < 0.0001), age differences across imaging modality groups were assessed using the Kruskal-Wallis test. Pairwise post-hoc comparisons were performed using Dunn’s test with Bonferroni correction. Categorical variables were reported as frequencies with percentages and compared using chi-square or Fisher’s exact tests as appropriate. Confidence intervals for proportions were calculated using Wilson score intervals to provide accurate estimates for small sample sizes. To adjust for potential selection bias in imaging modality assignment, Firth penalized logistic regression (bias-reduced maximum likelihood estimation with Jeffreys prior) was used to estimate adjusted odds ratios with 95% confidence intervals for the association between patient characteristics and multisystem disease, given the small number of outcome events. All statistical analyses were performed using StataNow 19.5 statistical software. Statistical significance was defined as *p* < 0.05.

### 2.4. Clinical Decision Algorithm Development

Based on study findings, a real-world clinical decision algorithm was developed incorporating statistically significant predictors of imaging selection and detection efficacy. The algorithm integrates patient age, initial disease location, radiation exposure, and cost considerations to select the optimal imaging modality. To provide preliminary internal validation of the key predictive component underlying the clinical decision algorithm, bootstrap validation (B = 1000 resamples) was performed for a logistic regression model predicting multisystem disease using imaging modality (PET vs. non-PET) and age as covariates. Model discrimination was quantified using the C-statistic and calibration was assessed using the Brier score and calibration slope with optimism correction applied to account for overfitting.

## 3. Results

### 3.1. Demographic Characteristics and Age-Related Imaging Selection Patterns

**Figure 1:**
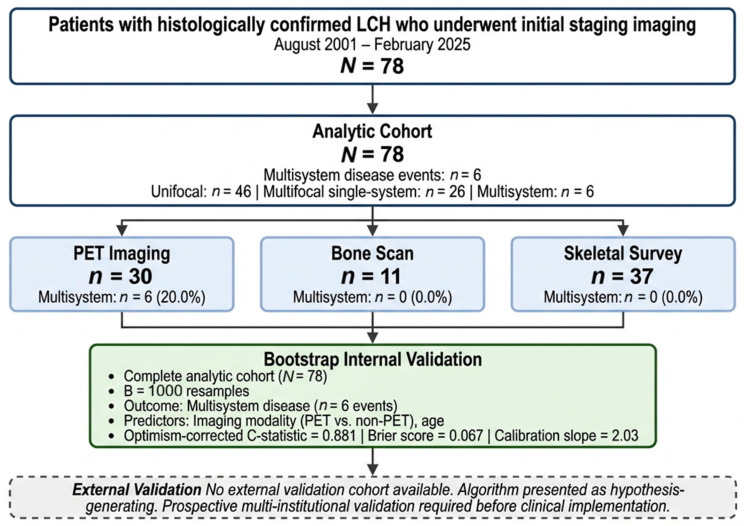
TRIPOD Flow Diagram. LCH, Langerhans cell histiocytosis: PET, positron emission tomography. The cohort consisted of 45 males (57.7%) and 33 females (42.3%), with a median age of 12.0 years (Interquartile range: [IQR]: 4.0–33.8 years). The disease extent at diagnosis included 46 patients (59.0%) with unifocal disease, 26 patients (33.3%) with multifocal disease involving a single system, and six patients (7.7%) with multisystem disease. Of these patients, 30 (38.5%; 95% CI 28.4–49.6%) underwent PET imaging, 11 (14.1%; 95% CI 8.1–23.5%) received bone scan evaluation, and 37 (47.4%; 95% CI 36.7–58.4%) were assessed with skeletal survey. Patient age differed significantly across imaging modality groups (Kruskal-Wallis H = 14.41, df = 2, *p* = 0.0007). Post-hoc pairwise testing with Bonferroni correction identified a significant difference between PET (median 34 years, IQR 8–56) and skeletal survey (median 7 years, IQR 2–14) (adjusted *p* = 0.0006). PET vs. bone scan (median 13 years, IQR 3–37; adjusted *p* = 0.542) and bone scan vs. skeletal survey (adjusted *p* = 0.367) did not reach statistical significance after correction.

The study cohort comprised 78 patients with biopsy-proven LCH who were evaluated at our institution between 2001 and 2025. The flow of participants through the study is presented in Figure 1.

**Figure 2:**
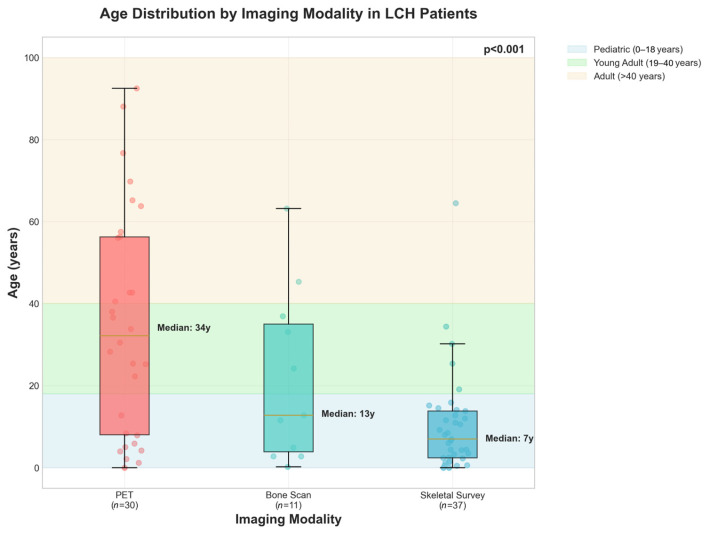
Age Distribution and Imaging Modality Selection. This box-and-whisker plot demonstrates age distribution for people who underwent PET (*n* = 30), bone scan (*n* = 11), or skeletal survey (*n* = 37). The boxes indicate the interquartile range, with the medians labeled for each group: 34 years for PET, 13 years for bone scan, and 7 years for skeletal survey.

Figure 2 displays the age distribution across imaging modalities, with individual patient data points, demonstrating a clear age stratification pattern. Skeletal surveys are predominantly utilized in pediatric patients, while PET is more commonly employed in adults.

### 3.2. Influence of Initial Disease Localization on Imaging Approach

The anatomic site of initial disease presentation significantly influenced the selection of imaging modality (*p* = 0.016). Among the 53 patients presenting with bone lesions, 28 (52.8%; 95% CI 39.7–65.6%) underwent skeletal survey, 15 (28.3%; 95% CI 18.0–41.6%) received PET imaging, and 10 (18.9%; 95% CI 10.6–31.4%) had bone scan (Table 1).

**Figure 3:**
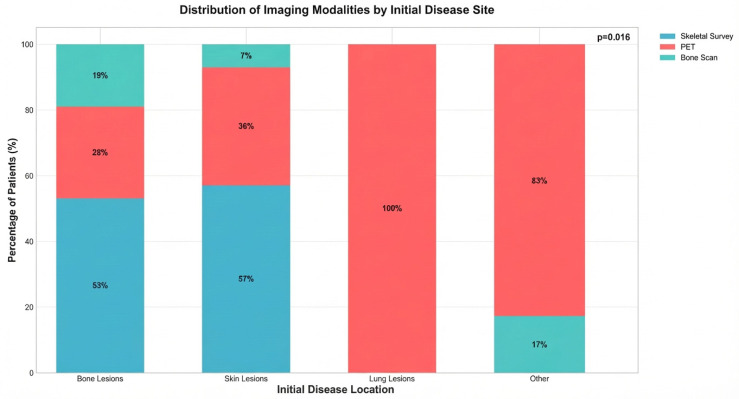
Initial Disease Location and Imaging Modality Selection. This bar plot shows the distribution of presenting initial disease site and the corresponding imaging modality selected. Skeletal surveys were most commonly used for bone and skin lesions, while lung lesions were exclusively evaluated with PET scans. ‘Other’ presenting location includes: isolated CNS involvement (*n* = 1), isolated lymph node involvement (*n* = 1), brain with axillary and inguinal lymphadenopathy (*n* = 1), colon/brain/vulva (*n* = 1), groin lymph node (*n* = 1), and lymph nodes of bilateral axilla, chest wall, and inguinal region (*n* = 1). PET imaging was utilized in 5 of 6 patients (83.3%; 95% CI 43.6–97.0%) in this subgroup; 1 of 6 patients (16.7%; 95% CI 3.0–56.4%) received skeletal survey. No bone scans were performed in this group.

Within the bone lesion subgroup, the skull was the most common site (21/53, 39.6%; 95% CI 27.6–53.1%), followed by long bones (18/53, 34.0%; 95% CI 22.7–47.4%), vertebrae (4/53, 7.5%; 95% CI 3.0–17.9%), pelvis (3/53, 5.7%; 95% CI 1.9–15.4%), and trunk (2/53, 3.8%; 95% CI 1.0–12.8%). For the 14 patients with cutaneous manifestations, 8 (57.1%; 95% CI 32.6–78.6%) underwent skeletal survey, 5 (35.7%; 95% CI 16.3–61.2%) received PET imaging, and 1 (7.1%; 95% CI 1.3–31.5%) was evaluated with bone scan. All 5 patients with pulmonary involvement were assessed exclusively with PET imaging (100%; 95% CI 56.6–100%). Among the 6 patients with other presenting locations, 5 (83.3%; 95% CI 43.6–97.0%) underwent PET and 1 (16.7%; 95% CI 3.0–56.4%) received skeletal survey, with no bone scans performed in this group (0%; 95% CI 0.0–39.0%). Figure 3 illustrates the distribution of imaging modalities stratified by anatomic site of initial disease presentation.

### 3.3. Disease Extent and Imaging Modality Selection Patterns

Analysis of disease extent showed distinct distributions of unifocal, multifocal, and multisystem involvement across imaging modalities. Multisystem disease was present in 6 of 78 patients (7.7%), and all six of these patients had undergone PET imaging. No multisystem cases occurred among patients staged with skeletal survey or bone scintigraphy (Fisher’s exact *p* = 0.006 for PET vs. skeletal survey; Bonferroni adjusted *p* = 0.018). Unifocal disease was observed in 16 of 30 PET patients (53.3%; 95% CI 36.1–69.8%), 7 of 11 bone scan patients (63.6%; 95% CI 35.4–84.8%), and 23 of 37 skeletal survey patients (62.2%; 95% CI 46.1–75.9%). Multifocal singlesystem disease was identified in 8 of 30 PET patients (26.7%; 95% CI 14.2–44.4%), 4 of 11 bone scan patients (36.4%; 95% CI 15.2–64.6%), and 14 of 37 skeletal survey patients (37.8%; 95% CI 24.1–53.9%). Table 2 presents the distribution of disease extent stratified by imaging modality, illustrating the relationship between disease complexity and the selection of advanced imaging.

### 3.4. Multivariable Predictors of Multisystem Disease

Firth penalized logistic regression was performed to evaluate independent predictors of multisystem disease after adjusting for age and presenting site. Age was not independently associated with multisystem disease (OR = 0.985 per year, 95% CI 0.946–1.026, *p* = 0.473). Presenting at an ‘other’ non-osseous location was independently associated with multisystem disease vs. bone presentation (OR = 15.6, 95% CI 1.65–148, *p* = 0.017). Lung involvement showed a non-significant trend (OR = 13.5, 95% CI 0.62–293, *p* = 0.098). Skin presentation was not independently associated with multisystem disease (OR = 2.40, 95% CI 0.29–20.1, *p* = 0.418). Given EPV = 2.0, these findings are interpreted as hypothesis-generating and require prospective validation.

### 3.5. Clinical Decision Algorithm

**Figure 4:**
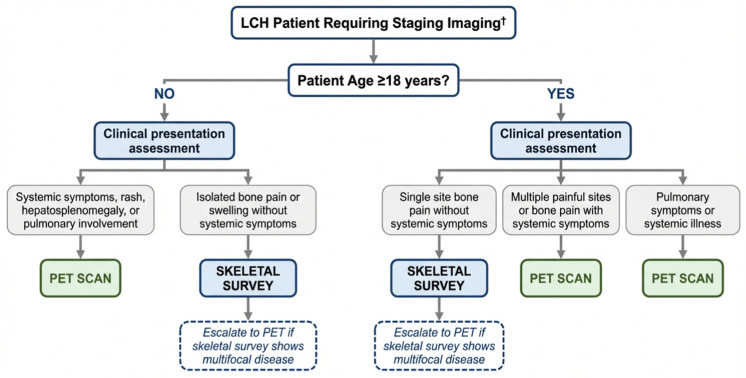
Comprehensive Clinical Decision Algorithm. This chart displays a real-world clinical decision algorithm that guides optimal imaging selection for patients with LCH. ^†^ This algorithm is hypothesis-generating and requires prospective external validation before clinical implementation. Bootstrap internal validation of the underlying logistic regression model yielded an optimism-corrected C-statistic of 0.881.

Based on these findings and the observed age and disease site selection patterns, we developed a real-world clinical decision algorithm incorporating patient age, initial disease location, and detection efficacy considerations to guide optimal imaging selection while balancing radiation exposure concerns (Figure 4).

### 3.6. Internal Validation of the Clinical Decision Algorithm

To provide preliminary internal validation of the logistic regression model underlying the algorithm’s core recommendation for PET in patients at risk for multisystem disease, bootstrap validation (B = 1000 resamples) was performed. The apparent C-statistic was 0.891; after optimism correction, the corrected C-statistic was 0.881 with a corrected Brier score of 0.067. The calibration slope of 2.03 reflects outcome sparsity (*n* = 6 multisystem events) rather than systematic miscalibration and should be interpreted accordingly. These metrics pertain specifically to the logistic model predicting multisystem disease and do not constitute direct validation of the full rule-based clinical decision algorithm presented in Figure 4, which incorporates additional clinical variables including symptom assessment and presenting site.

## 4. Discussion

In this 78-patient study of biopsy-proven LCH, we identified clinically significant associations between age, anatomic site, and the presence of systemic disease and imaging modality selection. First, older patients were significantly more likely to undergo PET imaging compared to both bone scans and skeletal surveys (*p* < 0.001). Second, the location of initial detection was significantly associated with imaging selection as individuals with bony lesions at the time of diagnosis were significantly more likely to undergo skeletal surveys compared to those with non-bone related diseases (*p* = 0.016). Finally, PET was the only modality associated with suspected multisystem disease detection in our cohort, consistent with clinicians’ tendency to reserve PET for patients with presentations concerning for systemic involvement.

Age-stratified PET utilization may reflect clinical decision-making that weighs the risks of radiation exposure against the benefits of diagnosis as the higher radiation dose associated with PET imaging (4–7 mSv) compared to skeletal survey (0.2–0.3 mSv) raises concerns in pediatric patients [21,24]. Despite these radiation concerns, multiple studies support the superior diagnostic accuracy of PET in pediatric LCH populations. Jessop et al. published a 2020 retrospective review of 109 scans in 33 patients (aged 7 weeks to 18 years), accurately identifying five recurrences in four patients with a false-positive rate of only 4% at initial diagnosis and 2% during follow-up [28]. These findings align with previous retrospective reviews, which demonstrate PET/CT’s superior accuracy in detecting lesions at initial diagnosis, higher sensitivity for identifying biopsy-proven disease, and enhanced capability for early detection of new lesions [29,30,31]. However, the enhanced ability of PET/CT to detect systemic disease, particularly in solid organs, does not necessarily translate to improved clinical outcomes or prognosis. While these mentioned studies demonstrate PET/CT has superior detection of extra-osseous lesions compared to conventional imaging, advanced imaging such as PET/CT does not necessarily improve overall survival in LCH because survival outcomes are primarily determined by disease biology and the effectiveness of systemic therapy, rather than by the sensitivity of imaging modalities used for staging or monitoring [5,18,32,33,34,35,36,37,38]. The primary clinical benefit appears to be treatment optimization rather than survival improvement due to PET/CT’s ability to distinguish metabolically active from inactive lesions, preventing unnecessary aggressive chemotherapy, potentially sparing patients from treatment-related morbidity [35,36]. However, this treatment precision benefit must be weighed against the cumulative radiation burden in pediatric patients requiring serial surveillance imaging. Given the lack of a clear survival advantage despite established superior diagnostic capabilities, the current age-stratified approach to PET utilization appears clinically appropriate, as it balances the documented benefits of treatment optimization against concerns regarding radiation exposure in this vulnerable population.

The cost differential between PET/CT and skeletal surveys in LCH evaluation represents a significant economic consideration, raising essential questions about the cost-effectiveness of LCH management. However, the financial justification may extend beyond baseline imaging costs when considering LCH-specific diagnostic challenges. PET identified all multisystem cases in our cohort reflecting a non-random pattern consistent with clinicians tending to select PET for patients with presentations suggestive of systemic involvement. The question now is whether this targeted use of advanced imaging meaningfully improves clinical decision-making given its higher cost and radiation burden. The cost differential becomes particularly problematic in pediatric populations, where our data show that skeletal surveys were used in 62.2% of cases, despite the availability of PET, suggesting that physicians are already considering the cost-benefit ratio in their decision-making. The economic burden is compounded by the need for serial imaging in LCH surveillance, which multiplies costs over the course of the disease. From a healthcare resource allocation perspective, repeat use of PET for serial imaging could significantly increase long-term costs for patients and health systems. Given that we observed PET utilization to be concentrated in older patients (median age 34 years) and specific clinical scenarios, such as pulmonary involvement, we suggest that selective rather than routine PET use may represent a more economically sustainable approach. Based on these economic considerations and the lack of demonstrated survival benefit, it may be more appropriate for PET to be used in pediatric populations to guide treatment optimization in suspected systemic disease.

Based on our findings and the observed clinical practice patterns, we propose a practical algorithm for imaging modality selection in LCH that balances diagnostic capability with cost and radiation considerations, utilizing pre-imaging clinical assessments that physicians can perform (Figure 4). The algorithm is structured around two sequential branch points: patient age and clinical symptom assessment. Notably, presenting site was independently associated with multisystem disease on multivariable analysis (OR = 15.6, *p* = 0.017), supporting the use of clinical symptom assessment (specifically the presence or absence of systemic symptoms, pulmonary involvement, and hepatosplenomegaly) as the primary driver of imaging selection within each age group. For adult patients (≥18 years), those with isolated bone pain and no systemic symptoms may be appropriately staged with a skeletal survey as a cost-effective first approach, while patients presenting with multiple symptomatic sites, systemic symptoms, or pulmonary involvement would undergo PET imaging. In adult patients, the lower radiation sensitivity relative to pediatric populations supports broader PET utilization, particularly in those presenting with multiple symptomatic sites or systemic symptoms where the probability of multisystem disease is higher. In cases of confirmed pulmonary involvement or overt systemic illness, PET imaging is the appropriate first-line modality regardless of age, given its established superior ability to characterize the full extent of disease burden and guide systemic therapy decisions. In pediatric patients, clinical symptom assessment similarly drives the initial imaging choice. A skeletal survey serves as the initial imaging approach for children presenting with isolated bone pain or swelling without systemic symptoms, particularly since bone lesions represented the most common presentation (67.9% of our cohort) and skeletal surveys were successfully utilized in 62.2% of pediatric cases. However, PET imaging is recommended for pediatric patients presenting with systemic symptoms, including fever, weight loss, rash, hepatosplenomegaly, or pulmonary involvement. In these cases, we observed that all pulmonary cases were staged with PET, and multisystem disease occurred exclusively among patients who underwent PET imaging compared with none in the skeletal survey group (Fisher’s exact *p* = 0.006). For cases where the initial skeletal survey reveals multifocal disease or concerning findings suggesting broader involvement, escalation to PET imaging is justified to guide treatment planning. This algorithm formalizes the clinician decision patterns observed in this cohort, reserving PET for presentations with higher pre-test concern for multisystem involvement. By aligning imaging selection with clinical presentation, it promotes consistency and resource stewardship while limiting unnecessary radiation exposure and cost.

Several strengths of this study should be noted. First, we provide the most extensive comparative analysis of imaging modalities in LCH to date, encompassing 78 biopsy-proven cases, which provides significant statistical power for this rare disease. Second, we analyzed real-world clinical practice patterns, providing practical insights applicable to routine patient care. Lastly, our analysis included patient age, disease location, extent, and imaging outcomes to provide a thorough understanding of staging decisions. However, the results presented in this study should be interpreted with consideration of several limitations that warrant further discussion. First, the imaging selection modality was not randomized and pulled from a single institution, leading to selection bias, as PET utilization was performed in patients with a higher suspicion of systemic disease, reflecting real-world practice patterns. As such, the findings are interpreted as hypothesis-generating rather than confirmatory. This purposeful clinical reasoning, however, was intentionally captured by this study to formalize a decision logic already being used effectively. Additionally, PET as an imaging modality has undergone considerable technological advancements throughout our study’s timeline, suggesting the detection rates presented in this study may represent conservative estimates of PET’s current diagnostic capabilities [39]. Despite promising results seen in contemporary studies with the use of PET/MRI or WB MRI, our study did not evaluate these modalities as they were not used at our institution during the study period. Furthermore, the small number of multisystem disease events (*n* = 6) yields an events-per-variable ratio of 2.0 for the Firth logistic regression model precluding definitive multivariable inference and mandating that regression findings be interpreted as hypothesis-generating. Internal bootstrap validation of this model yielded an optimism-corrected C-statistic of 0.881 and Brier score of 0.067, providing preliminary evidence of discriminative performance. However, the elevated calibration slope (2.03) reflects outcome sparsity rather than model stability. The clinical decision algorithm presented in Figure 4 integrates these findings alongside additional clinical variables including presenting site and symptom assessment, and represents a practical evidence-informed framework derived from real-world practice patterns at a high-volume tertiary center. Prospective multi-institutional validation in larger cohorts would further strengthen the algorithm’s generalizability and support broader clinical implementation. Lastly, we did not conduct head-to-head assessments between imaging modalities to directly assess their performance in detecting multisystem diseases. Despite these limitations, we believe our findings reflect real-world clinical practice patterns and provide meaningful evidence to guide imaging selection. Further studies incorporating prospective randomized comparisons and formal cost-effectiveness analyses would provide more substantial evidence for optimal imaging protocols in LCH management.

## 5. Conclusions

This retrospective analysis of 78 patients with histologically confirmed LCH demonstrates that the initial imaging modality selection is significantly influenced by patient age, the anatomic site of initial disease presentation, and clinical suspicion of systemic involvement. While PET imaging demonstrates superior capability for detecting multisystem disease, its enhanced diagnostic sensitivity must be balanced against substantially higher costs and radiation exposure, particularly in pediatric populations. Our proposed clinical decision algorithm provides evidence-informed guidance, recommending a skeletal survey as the first-line imaging modality for patients with isolated bone symptoms, while reserving PET for cases with systemic symptoms or pulmonary involvement. This selective approach optimizes diagnostic accuracy while minimizing unnecessary costs and radiation exposure in this vulnerable population.

## Figures and Tables

**Table 1:** Baseline demographic and clinical characteristics stratified by imaging modality.

Characteristic	PET (*n* = 30)	Bone Scan (*n* = 11)	Skeletal Survey (*n* = 37)	Total (*n* = 78)	*p*-Value
Age, median (IQR)	34 (8–56)	13 (3–37)	7 (2–14)	12 (4–34)	0.0007
Sex, *n* (%)					0.075
Male	15 (50.0%)	4 (36.4%)	26 (70.3%)	45 (57.7%)	
95% CI	33.2–66.8%	15.2–64.6%	54.2–82.5%	46.6–68.0%	
Female	15 (50.0%)	7 (63.6%)	11 (29.7%)	33 (42.3%)	
95% CI	33.2–66.8%	35.4–84.8%	17.5–45.8%	32.0–53.4%	
Presenting Location, *n* (%)					0.016
Bone	15 (50.0%)	10 (90.9%)	28 (75.7%)	53 (67.9%)	
95% CI	33.2–66.8%	62.3–98.4%	59.9–86.6%	57.0–77.3%	
Skin	5 (16.7%)	1 (9.1%)	8 (21.6%)	14 (17.9%)	
95% CI	7.3–33.6%	1.6–37.7%	11.4–37.2%	11.0–27.9%	
Lung	5 (16.7%)	0 (0.0%)	0 (0.0%)	5 (6.4%)	
95% CI	7.3–33.6%	0.0–25.9%	0.0–9.4%	2.8–14.1%	
^†^ Other	5 (16.7%)	0 (0.0%)	1 (2.7%)	6 (7.7%)	
95% CI	7.3–33.6%	0.0–25.9%	0.5–13.8%	3.6–15.8%	

IQR, interquartile range; PET, positron emission tomography. Age compared using Kruskal-Wallis test; categorical variables compared using chi-square or Fisher exact test as appropriate. All proportions reported with Wilson score 95% confidence intervals. ^†^ ‘Other’ presenting location includes: isolated CNS involvement (*n* = 1), isolated lymph node involvement (*n* = 1), brain with axillary and inguinal lymphadenopathy (*n* = 1), colon/brain/vulva (*n* = 1), groin lymph node (*n* = 1), and lymph nodes of bilateral axilla, chest wall, and inguinal region (*n* = 1). PET imaging was utilized in 5 of 6 patients (83.3%; 95% CI 43.6–97.0%) in this subgroup.

**Table 2:** Disease Extent at Diagnosis by Imaging Modality.

Disease Extent	PET (*n* = 30)	Bone Scan (*n* = 11)	Skeletal Survey (*n* = 37)	Total (*n* = 78)
Unifocal, *n* (%)	16 (53.3%)	7 (63.6%)	23 (62.2%)	46 (59.0%)
95% CI	36.1–69.8%	35.4–84.8%	46.1–75.9%	
Multifocal Single-System, *n* (%)	8 (26.7%)	4 (36.4%)	14 (37.8%)	26 (33.3%)
95% CI	14.2–44.4%	15.2–64.6%	24.1–53.9%	
Multisystem, *n* (%)	6 (20.0%)	0 (0.0%)	0 (0.0%)	6 (7.7%)
95% CI	9.5–37.3%	0.0–25.9%	0.0–9.4%	
*p*-value	*p* = 0.032 (χ^2^ = 10.52, df = 4; permutation *p* = 0.029, B = 10,000) ^†^			

This table demonstrates that PET scans are most frequently utilized for multisystem disease, while skeletal survey and bone scans were more frequently used for patients with unifocal disease. Minimum expected cell count = 0.85; permutation-based *p*-value reported given sparse cells. Pairwise Fisher’s exact tests for multisystem disease: PET vs. skeletal survey *p* = 0.006 (Bonferroni-adjusted *p* = 0.018); PET vs. bone scan *p* = 0.167 (adjusted *p* = 0.502); bone scan vs. skeletal survey *p* = 1.000. All proportions reported with Wilson score 95% confidence intervals. ^†^ Chi-square test with permutation correction (B = 10,000 resamples). 95% CIs calculated using the Wilson score method. PET, positron emission tomography.

## Data Availability

The dataset supporting the conclusions of this article is not publicly available due to patient privacy concerns, including HIPAA regulations and institutional policy. However, it may be available from the corresponding author on reasonable request and with appropriate institutional approval. Summary data supporting the findings of this study are included within the article.

## Abbreviations

The following abbreviations are used in this manuscript:

LCH	Langerhans Cell Histiocytosis
PET	Positron Emission Tomography
IQR	Interquartile Range
EG	Eosinophilic Granuloma
ANOVA	Analysis of Variance
